# Differences in Anti-Inflammatory Actions of Intravenous Immunoglobulin between Mice and Men: More than Meets the Eye

**DOI:** 10.3389/fimmu.2015.00197

**Published:** 2015-04-28

**Authors:** Angela S. W. Tjon, Rogier van Gent, Teunis B. Geijtenbeek, Jaap Kwekkeboom

**Affiliations:** ^1^Department of Gastroenterology and Hepatology, Erasmus MC-University Medical Center, Rotterdam, Netherlands; ^2^Department of Experimental Immunology, Academic Medical Center, Amsterdam, Netherlands

**Keywords:** IVIg, anti-inflammatory, autoimmunity, sialylation, regulatory T cells, Fcγ receptors

## Abstract

Intravenous immunoglobulin (IVIg) is a therapeutic preparation of polyspecific human IgGs purified from plasma pooled from thousands of individuals. When administered at a high dose, IVIg inhibits inflammation and has proven efficacy in the treatment of various autoimmune and systemic inflammatory diseases. Importantly, IVIg therapy can ameliorate both auto-antibody-mediated and T-cell mediated immune pathologies. In the last few decades, extensive research in murine disease models has resulted in the elucidation of two novel anti-inflammatory mechanisms-of-action of IVIg: induction of FcγRIIB expression by sialylated Fc, and stimulation of regulatory T cells. Whereas controversial findings in mice studies have recently inspired intense scientific debate regarding the validity of the sialylated Fc-FcγRIIB model, the most fundamental question is whether these anti-inflammatory mechanisms of IVIg are operational in humans treated with IVIg. In this review, we examine the evidence for the involvement of these anti-inflammatory mechanisms in the therapeutic effects of IVIg in humans. We demonstrate that although several elements of both immune-modulatory pathways of IVIg are activated in humans, incorrect extrapolations from mice to men have been made on the molecular and cellular components involved in these cascades that warrant for critical re-evaluation of these anti-inflammatory mechanisms of IVIg in humans.

## Introduction

Intravenous immunoglobulin (IVIg) was initially administered to restore humoral immunity in patients with primary or secondary immunodeficiency, as it contains a wide spectrum of antibody specificities, representative for the natural antibody repertoire of the adult human population. After it had been shown that high-dose IVIg treatment (fourfold higher than supplementation dose) (1) could ameliorate idiopathic thrombocytopenic purpura (ITP) (2), its anti-inflammatory properties have increasingly been exploited to treat various autoimmune and systemic inflammatory diseases.

Several non-exclusive mechanisms by which IVIg exerts its anti-inflammatory effects have been elucidated over the past few decades. These include neutralization of autoantibodies by anti-idiotype interactions, increased clearance of pathogenic antibodies by saturation of the neonatal FcR (FcRn), prevention of binding of pathogenic immune complexes (ICs) to activating Fcγ-receptors (FcγR), modulation of FcγR expression, inhibition of the complement cascade, reduced pro-inflammatory cytokine production, inhibition of dendritic cells (DCs) and B cells, inhibition of T-helper (Th)1 and Th17 differentiation, and expansion and enhanced suppressive function of CD4^+^FOXP3^+^ regulatory T cells (Tregs) (1, 3–7).

It is important to realize that most of the anti-inflammatory mechanisms of IVIg have been elucidated in murine studies. Of course, studies using animal models are ideal to determine causal relationships between the elements of anti-inflammatory cascades. However, it is actually surprising that validation of these mechanistic findings in human patients treated with IVIg is severely lacking. Extrapolation of the anti-inflammatory mechanisms from murine studies to humans treated with IVIg is by no means trivial as fundamental differences between the murine and human immune system, as well as the xenobiotic nature of human IgGs when administered to a different organism, are likely to affect the mode-of-action of IVIg within these species.

In this review, we investigate the similarities and differences between the anti-inflammatory mechanisms activated by IVIg in mice and humans. We will focus on the two most advertised anti-inflammatory mechanisms in recent years: induction of FcγRIIB expression by sialylated Fc and stimulation of Tregs. We examine the evidence for the involvement of their proposed cellular and molecular components in immunomodulation by IVIg therapy in humans and, when validation in humans is still lacking or incomplete, discuss their translatability from murine to human studies by taking the biology of both species into account.

## Sialylated IVIg and FcγRIIB Upregulation

In the last decade, landmark studies have revealed that IgGs with α2,6-sialic acid-containing N-linked glycans attached to the IgG Fc (sFc) display potent anti-inflammatory activity in antibody-mediated inflammation in experimental animal models. Identification of the anti-inflammatory properties of this IgG fraction started with a study in a murine ITP-model in which the protective effects of IVIg appeared dependent on (1) upregulation of FcγRIIB expression on effector macrophages, thereby limiting IC-mediated activation, and (2) the IgG Fc (8). In subsequent studies, the protective effect of IVIg in a mouse model of IC-mediated (K/BxN) arthritis was shown to be mediated by colony stimulating factor-1 (CSF-1)-dependent macrophages that act as sensors for IVIg and are involved in the induction of inhibitory FcγRIIB expression on CSF-1-independent effector macrophages, thereby raising the threshold for activation of these cells by auto-antibody-IC (9). These CSF-1-dependent IVIg-sensoring macrophages were identified as splenic SIGN-R1^+^ marginal zone macrophages (MZM) which were able to bind sFc (10).

**Figure 1:**
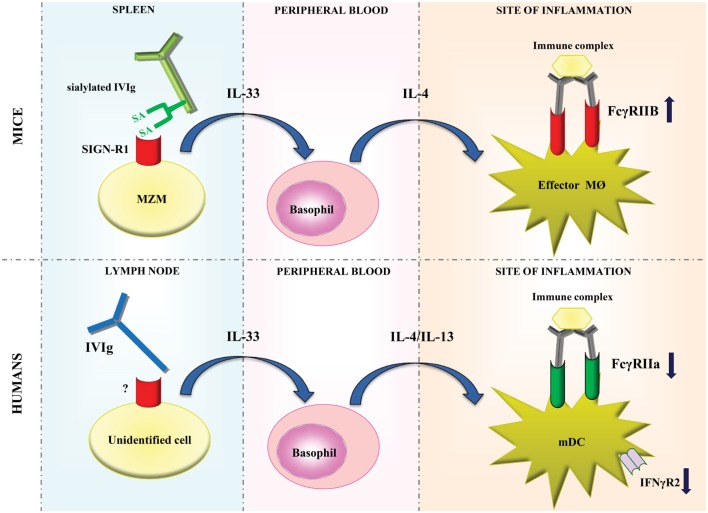
**Models of modulation of FcγR expression by IVIg in mice and humans**. In mice, sialylated IVIg is hypothesized to bind to SIGN-R1 expressed on splenic marginal zone macrophages and induce IL-33 production. IL-33 subsequently promotes the production of IL-4 by basophils, which enhances the expression of FcγRIIB expression on effector macrophages at the site of inflammation. In humans, IVIg may bind to an, as of yet, unidentified cell type which probably resides in lymph nodes. This is hypothesized to induce IL-33 production, which in turn enhances IL-4 and IL-13 production by basophils resulting in decreased expression of FcγRIIa and IFNγR2 on myeloid dendritic cells. MZM, marginal zone macrophages; SA, sialic acid; MØ, macrophage; mDC, myeloid dendritic cell.

Parallel studies demonstrated that sFc was essential for the anti-inflammatory activity of IVIg in the K/BxN arthritis model in a FcγRIIB-dependent manner (11–13). Infusion of sFc protected wild type, but not SIGN-R1^−/−^ mice from arthritis, suggesting that binding of sFc to SIGN-R1 on MZM was required for the anti-inflammatory effect (10). A human ortholog of SIGN-R1, DC-SIGN, was also able to bind sFc *ex vivo* (10), and the protective activity of sFc and sialylated IVIg (sIVIg) was retained upon induction of arthritis in SIGN-R1^−/−^ mice that transgenically expressed human DC-SIGN (13). These data suggested that DC-SIGN might be able to mediate the anti-inflammatory properties of sIVIg in humans *in vivo*. In addition, it was shown that sIVIg induced the production of IL-33 in the spleen of wild type but not SIGN-R1^−/−^ mice. IL-33 subsequently promoted expansion of basophils in the circulation and stimulated their production of IL-4 and IL-13, which enhanced the expression of FcγRIIB expression on macrophages and monocytes, thereby providing a link between IVIg-sensor macrophages and induction of FcγRIIB expression on myeloid effector cells (Figure 1) (13).

The elucidation of this mechanistic model, that is founded on the absolute requirement of sFc and FcγRIIB expression, seemed to profoundly improve our understanding of the protective effects of IVIg in antibody-mediated inflammation in mice. However, due to findings in other murine studies on IVIg, intense scientific debate regarding the validity of this anti-inflammatory model has commenced. Upregulation of inhibitory FcγRIIB expression by IVIg has been demonstrated in various animal models as an effector mechanism by which antibody-mediated immune diseases are prevented (8, 9, 14). In contrast, in murine studies on ITP (15, 16) and experimental autoimmune encephalomyelitis (EAE) (17), the beneficial effect of IVIg was also observed in FcγRIIB^−/−^ mice, challenging the absolute requirement for FcγRIIB in IVIg-treated mice. In addition, various recent reports question whether Fc-sialylation is absolutely required for the anti-inflammatory effects of IVIg in mice (18). In several studies on murine models of ITP (19, 20) [in contrast to other studies on ITP reporting an indispensable role for sialylation (11, 14, 21)], EAE (22), arthritis (23), and herpes simplex virus (HSV)-induced encephalitis (24), the anti-inflammatory properties of IVIg were sialylation-independent. In addition, the contribution of the IgG Fc-part to the anti-inflammatory effects of IVIg has been questioned in a study showing that the protective effects of IVIg in a mouse EAE model were F(ab′)_2_-dependent (17).

A matter that may complicate this debate is that in most murine studies IVIg was administered prophylactically, while in humans IVIg is given after a disease has established. This difference complicates translation of insights from murine studies to humans. A recent comparison between prophylactic and therapeutic IVIg treatments in several murine disease models (ITP, arthritis and skin-blistering disease) has shown that the anti-inflammatory effects of IVIg were similar, and were dependent on FcγRIIB expression and sialylation, although SIGN-R1 was not essential in all disease models when IVIg was administered therapeutically (14). These data suggest that there is considerable overlap between the anti-inflammatory activities of IVIg in mice upon prophylactic or therapeutic administration, but it would be helpful if future murine studies concentrated on the anti-inflammatory activities of therapeutic IVIg administration only.

A recent study by Washburn et al. reported that some of the discrepancies between studies on sIVIg or sialylated Fc may be caused by differences in the protocols used to generate and/or purify sialylated or desialylated IVIg/Fc. Commonly used sialyl-transferase enzyme preparations were found be contaminated with other glycolytic enzymes that catalyzed undesired modifications in the enzymatically produced sialylated IgG/Fc products. By establishing industrial-scale protocols and quality control steps, Washburn et al. enzymatically generated tetra Fc-sialylated IVIg (s4-IVIg; the glycans on each of the two heavy chains terminate in two sialic acid residues) that was devoid of undesired modifications in the glycan structures. When administered in four different murine disease models, s4-IVIg was as effective as conventional IVIg, but at a 10-fold lower dose. This was apparent when s4-IVIg was given prophylactically as well as therapeutically. It has to be noted though that therapeutic and prophylactic IVIg treatments were not compared in the same disease model (25). In addition, this study does not refute the data from other published studies in which treatment with desialylated IVIg was still as effective as treatment with conventional IVIg (22, 23).

Regardless of these contradictory findings, the most fundamental question now is whether there is any evidence that a similar mechanism mediates the anti-inflammatory effects of high-dose IVIg-therapy in humans. In the following sections, we will show in a balanced account that there is indeed evidence, although limited and circumstantial, for activation of this anti-inflammatory pathway by IVIg in humans. However, due to biological differences between mice and men, a number of issues regarding the anti-inflammatory actions of IVIg in humans, such as involvement of FcγRIIb modulation, splenic MZM, DC-SIGN, and the dependence on sFc, need to be critically reconsidered.

### IVIg and FcγIIb expression in humans

Is there any evidence for the increase of FcγRIIb expression in humans treated with IVIg? Recently, we found no increase of FcγRIIb expression on circulating monocytes and DCs in patients treated with high-dose IVIg for diverse autoimmune pathologies (26). These data corroborate findings in IVIg-treated patients with common variable immunodeficiency (CVID) which showed that *Fc*γ*RIIb* mRNA expression of circulating monocytes did not change upon low-dose IVIg treatment (27). Two other studies on patients with ITP and Kawasaki disease also showed no upregulation of FcγRIIb expression on monocytes after IVIg infusion, however, the validity of these results can be questioned as the antibody used to detect FcγRIIb in these studies binds to an intracellular epitope of the protein while no permeabilization protocol was applied (28, 29). In *in vitro* studies, IVIg did not induce upregulation of FcγRIIb expression on human myeloid DCs (26, 30). These findings seemed to be corroborated in a recent study that showed no modulation of FcγRIIb expression by IVIg on human macrophages *in vitro*. However, in this study IVIg was added to the cultures at a concentration at least 100-fold lower than the concentration required to reflect the increment in IgG levels observed upon high-dose IVIg treatment *in vivo* (10 mg/ml) (31). In contrast to these studies, a majority of patients with chronic inflammatory demyelinating polyneuropathy (CIDP) showed increased expression of FcγRIIb on monocytes and B cells after IVIg treatment (32). It has to be noted that the untreated CIDP patients in this study showed reduced FcγRIIb expression and the observed increase may have reflected a normalization of FcγRIIb expression levels upon reduction of overall inflammation by IVIg therapy. So strikingly, whereas IVIg treatment in several murine studies has shown to stimulate expression of inhibitory FcγRIIB on myeloid cells (8, 9, 24), most evidence in humans shows that FcγRIIb expression is not affected by IVIg, although these findings need to be extended in independent studies without technical issues.

Do these observations therefore imply that modulation of FcγR expression is not involved in the anti-inflammatory effects of IVIg treatment in humans? Although we did not find increase of FcγRIIb expression after high-dose IVIg treatment in patients with autoimmune diseases, we did find downregulation of another FcγR, the activating FcγRIIa, on circulating myeloid DCs (26). Given the differences in expression of FcγRs between mice and men, it is not surprising that the effects of IVIg treatment on FcγR modulation in mice are distinct from those in humans. Humans have six different FcγRs, namely FcγRIa, FcγRIIa, FcγRIIb, FcγRIIc, FcγRIIIa, and FcγRIIIb, while mice have four: FcγRI, FcγRIIB, FcγRIII, and FcγRIV (33–35). Thus, FcγRIIa, which we showed to be downregulated by IVIg treatment, is not present in mice.

In addition to downregulation of FcγRIIa expression on circulating myeloid DCs, we observed an increase in plasma levels of IL-33 and the Th2 cytokines IL-4 and IL-13 upon high-dose IVIg treatment, showing homology between the anti-inflammatory activity of IVIg in mice and men (26). Enhanced IL-33 plasma levels were also observed in another study in a cohort of autoimmune disease patients treated with IVIg, although IL-4 in plasma of these patients was hardly detectable and no expansion of basophils in peripheral blood of these patients was observed (36). *In vitro* experiments on human myeloid DCs suggested that FcγRIIa downregulation after IVIg treatment is not directly caused by IVIg, but rather indirectly by the elevated levels of IL-4 and IL-13, and resulted in suppressed responses of myeloid DCs to IC-stimulation (26).

Thus, IVIg therapy downregulates expression of the activating FcγRIIa in humans, instead of upregulation of the inhibitory FcγRIIB as was observed in mice (Figure 1). Interestingly, we found that activation of the cytokine cascade involving IL-33 and the Th2 cytokines IL-4 and IL-13 by IVIg is shared by mice and men. In addition, we found that these cytokines also downregulate expression of the IFN-γ receptor 2 subunit on myeloid DCs in humans (26), which may contribute to suppression of cellular immunity by IVIg (Figure 1) (37).

In a study by Siragam et al., it was shown that IVIg could also confer its anti-inflammatory effects by affecting signaling through activating FcγRs. In a mouse model of ITP, IVIg ameliorated the disease by directly interacting with activating FcγRs, but not the inhibitory FcγRIIB, on DCs. Strikingly, adoptive transfer of *ex vivo* IVIg-treated DCs was able to ameliorate ITP. These data suggest that IVIg forms soluble ICs *in vivo* that prime dendritic-cell regulatory activity (38). This may be not entirely surprising as it is likely that the infused IgGs are able to bind to xenogeneic polymorphic murine proteins, resulting in the formation of soluble ICs. Although intriguing, there is to date no evidence that a similar mechanism of action of IVIg occurs in humans.

### IVIg-sensing cell types in human lymphoid organs

Since high-dose IVIg induces the production of IL-33 and Th2 cytokines IL-4 and IL-13 in humans, the question emerges by which cell types these cytokines are produced. It is likely that, as in mice, basophils are the cellular source of IL-4 and IL-13 production after IVIg treatment in humans. Several *in vitro* studies have shown that human basophils produce IL-4 and IL-13 upon stimulation with IL-33 (39–41). The cellular source responsible for IL-33 production upon IVIg treatment however differs between mice and men. In murine studies, it has been demonstrated that splenic MZM are important in initiating the IL-33-Th2 cytokine cascade upon IVIg administration, as the protective effect of IVIg was lost after splenectomy or specific depletion of MZM. Therefore, MZM were suggested to be responsible for binding and initiating the protection mediated by sIVIg or sFc via SIGN-R1 (9, 10). It is important to note though, that it has not yet been formally proven that MZM are the source of IL-33 production upon IVIg treatment in mice. Importantly, the human and murine spleen differ to a major extent, both in morphology and the presence of specific cell types. MZM are diffusely spread within the marginal zone of the murine spleen (42), but they are not present in the human spleen (43). In comparison to the murine spleen, the human spleen contains an additional zone, called the perifollicular zone, which is located between the marginal zone and the red pulp (44). It contains a special subset of macrophages, of which some express DC-SIGN (45). Although their anatomical location in the human spleen is different, it can be speculated that these perifollicular macrophages are the human counterparts of murine MZM and can produce IL-33 upon IVIg treatment.

However, arguing against an indispensable role for the spleen in mediating the anti-inflammatory effects of IVIg in humans is the observation that IVIg is still an effective anti-inflammatory treatment for splenectomized ITP patients (46). This observation suggests that in humans either IL-33 production is dispensible for the anti-inflammatory effect of IVIg therapy or cell type(s) outside the spleen can produce IL-33 upon IVIg treatment. Indeed, IVIg induces *IL-33* mRNA expression in primary human lymph node cells *in vitro* (26) but not in human splenocytes (26, 36). In human lymph nodes, medullary sinus macrophages (MSM) as well as subcapsular sinus macrophages (SSM) are likely candidates for IVIg-induced IL-33 production *in vivo* as they express DC-SIGN (47, 48). Although we were not yet able to determine whether MSM an SSM are the cellular sources of IL-33, we did establish that human macrophages produce IL-33 upon IVIg exposure *in vitro* (26). Epithelial cells and fibroblasts can also produce IL-33 and may be alternative cellular sources (49, 50).

Recent studies using two different murine models of ITP showed that splenectomy in mice did not impair the protective effect of IVIg (15, 21). In one of these models, the protective effect of IVIg was even independent of IL-33 and IL-4 signaling, although still dependent on SIGN-R1 and sFc (21). This suggests that, as in humans, cells at anatomical locations outside the spleen may alternatively mediate the anti-inflammatory properties of sIVIg in mice.

Collectively, these data indicate the need to study which human tissues and cells are involved in stimulation of IL-33 production by IVIg. Obviously, availability of tissues from IVIg-treated patients is limited, but *in vitro* studies using human tissues and cells can help to clarify which human cell types are able to bind IVIg and produce IL-33.

### Involvement of DC-SIGN in the anti-inflammatory activity of IVIg in humans

The evidence that DC-SIGN might be involved in the anti-inflammatory response to IVIg in humans is derived from the observation that DC-SIGN can replace its murine ortholog SIGN-R1 in IL-33-mediated protection from serum-induced arthritis in mice upon sFc treatment (13). However, *in vitro* studies using human DC-SIGN expressing cells did not provide a clear indication for involvement of DC-SIGN in the protective effects of IVIg. We found that induction of IL-33 production in human macrophages by IVIg was not inhibited by blocking DC-SIGN (26). Moreover, human splenocytes and monocyte-derived DCs (moDCs), that both abundantly express DC-SIGN, did not produce IL-33 upon exposure to IVIg (26, 36). Currently, the only study showing a role for DC-SIGN in mediating an immune-modulatory effect of IVIg on human cells comes from an *in vitro* study in which it was shown that DC-SIGN was partially responsible for the IVIg-mediated induction of Tregs by prostaglandin E2 (PGE2)-producing moDCs. However, this effect was not Fc- but F(ab′)_2_-dependent and therefore not mediated by sFc binding to DC-SIGN (51).

Whether DC-SIGN is the proper human homolog of SIGN-R1 may be argued. L-SIGN, another human C-type lectin, also shares homology to SIGN-R1 with regard to cellular expression pattern. SIGN-R1 and L-SIGN are both expressed by liver sinusoidal and lymph node endothelial cells (52–54). Scattered cells expressing L-SIGN were also observed in the human splenic perifollicular zone which, as suggested above, might represent the human counterparts to splenic SIGN-R1^+^ MZM in mice (13). Shared human DC-SIGN and murine SIGN-R1 expression has been observed for MSM in lymph nodes (42, 48) and, likely similar to L-SIGN, on some macrophages in the perifollicular zone of the human spleen (45). However, DC-SIGN is also expressed on subsets of human monocytes (13), myeloid DCs (53), and SSM in human lymph nodes (47, 48), which are cell types in mice that do not express SIGN-R1. L-SIGN is able to bind to sFc *in vitro*, although with reduced affinity compared to DC-SIGN, which suggests a possible role of L-SIGN in sensing sIVIg in humans. In our opinion, the published results of selective blockade of DC-SIGN in SIGN-R1^−/−^ mice that express a human transgene containing both DC-SIGN and L-SIGN, do not completely exclude involvement of L-SIGN in the anti-inflammatory effects of sFc (13). Additional studies using human cells are required to demonstrate whether L-SIGN and/or DC-SIGN are involved in mediating anti-inflammatory effects of IVIg in humans.

Whether DC-SIGN can bind sIVIg or sFc with sufficient affinity is still a matter of debate. It does not bind sialylated glycans or glycoconjugates (55–57), suggesting that binding of sFc by this lectin must involve non-canonical interactions. A recent study suggested that sialylation of the N-linked Fc glycan structurally affects the IgG Cγ2 domain, causing a so-called “closed” state of sFc which would enable interaction with DC-SIGN, while asialylated Fc has an “open” state, resulting in preferential binding to FcγRs (58). However, this model of sFc-DC-SIGN interaction is controversial and has initiated an ongoing scientific debate as contradictory evidence shows that Fc sialylation does not induce alterations in the Fc conformation (59, 60). Moreover, binding affinity of engineered IgG glycoforms that were either hyper-α2,6-sialylated, asialylated or deglycosylated to tetramerized DC-SIGN was shown not to differ and, strikingly, was Fab- but not Fc-dependent (61).

Taken together, the evidence for a prominent role of DC-SIGN in mediating the anti-inflammatory activity of IVIg in humans is very limited. Moreover, when considering recent murine studies on IVIg, it was shown that SIGN-R1 was dispensable for therapeutic amelioration of ITP as well as prevention of antigen-driven airway disease in mice upon treatment with IVIg (14, 62), thereby questioning whether SIGN-R1 is absolutely required to confer the anti-inflammatory properties of IVIg in mice. As we will discuss in the following section, the requirement for sFc which was found to confer the anti-inflammatory activity of IVIg via DC-SIGN, is even more heavily debated.

### Contribution of Fc sialylation to the anti-inflammatory activity of IVIg in humans

The only data available on the contribution of sIVIg to the anti-inflammatory effects of IVIg on human immune cells are from a study showing that sIVIg, but not asialyated IVIg, stimulated apoptosis in human B cells *in vitro* via CD22 (see also Alternative IVIg-Sensing Molecules) (63). However, it seems unlikely that these immunomodulatory effects were mediated by sFc, as in this study sIVIg was enriched by *Sambucus nigra agglutinin* (SNA) lectin fractionation which mainly enriches F(ab′)_2_-sialylated IgG (20, 64). Interestingly, infusion of Fc fragments for treatment of childhood ITP was effective, suggesting that the IgG Fc is involved in the anti-inflammatory effects of IVIg in humans (65, 66). However, inhibition of human monocyte function by IVIg was F(ab′)_2_-dependent (64). In addition, F(ab′)_2_ fragments, but not Fc fragments, were shown to mediate Treg expansion via human moDC (51).

As previously mentioned, these often paradoxical results have led to an ongoing scientific debate on the importance of Fc-sialylation and IgG Fc in the anti-inflammatory activity of IVIg (3). Currently, there is no data showing that specific IgG glycoforms are required for the beneficial effects of IVIg in humans. Studies in which the effects of sIVIg and sFc on human immune cells are compared to those of non-sialylated IVIg and non-sialylated Fc are therefore highly required.

### Alternative IVIg-sensing molecules

Despite the contradictory findings on the requirement of Fc sialylation, there are reports that do show a role for sIVIg in relation to two other sialic acid-binding proteins, namely the C-type lectin dendritic cell immunoreceptor (DCIR) and the sialic acid-binding Ig-like lectin (Siglec) CD22. Murine DCIR was shown to specifically bind sIVIg *in vitro*, and DCIR-expressing tolerogenic DCs-induced expansion of Tregs which attenuated ovalbumin-induced airway hyperresponsiveness in mice in a FcγR- and SIGN-R1-independent manner. Moreover, IVIg-induced DCIR expression on DCs, thereby propagating its anti-inflammatory activity (62). Due to its recent discovery, it has not been explored yet whether DCIR is involved in the anti-inflammatory effects of IVIg in humans.

CD22 (Siglec-2) is expressed on murine and human B cells and murine DCs (67), and binds α2,6-sialic acid-containing glycans with high specificity (68). sIVIg, but not asialyated IVIg, was shown to bind to CD22 on human B cells *in vitro*, resulting in reduced BCR signaling and enhanced apoptosis upon stimulation with anti-IgM (63).

**Figure 2:**
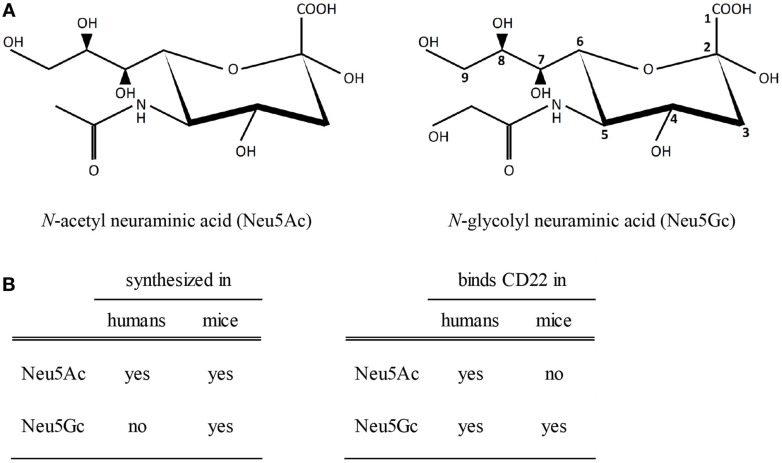
**Sialic acids and binding to CD22**. **(A)** A schematic view of *N*-acetylated and *N*-glycolylated sialic acid structures. The carbon at position 5 is either *N*-acetylated (left) or *N*-glycolylated (right). **(B)** Biosynthesis of sialic acids in humans and mice and binding properties of sialic acids to human or murine CD22.

Arguing against a role for CD22 in mediating the anti-inflammatory effects of IVIg is the observation that CD22^−/−^ mice were still protected from ITP and serum-induced arthritis by IVIg (69). However, it should be noted that murine studies on the involvement of CD22 in the effects of sIVIg are not valid due to differences between sialic acids in humans and mice. Sialic acids are nine-carbon sugars which contain a carbon ring (C2–C6) and an exocyclic side chain (C7–C9). One group of sialic acids, called neuraminic acids, are *N*-acetylated at C5 in the carbon ring, yielding *N*-acetyl neuraminic acid (Neu5Ac) (Figure 2A). In many vertebrates, including mice, this *N*-acetyl group can be converted by the enzyme CMP-*N*-acetyl hydroxylase (CMAH) to a *N*-glycolyl group resulting in *N*-glycolyl neuraminic acid (Neu5Gc). Importantly, CMAH is non-functional in humans (70). Therefore, terminal sialic acids attached to N-linked glycans like those in human IgG molecules are Neu5Ac and not Neu5Gc. While human CD22 binds both Neu5Ac and Neu5Gc, murine CD22 can only bind Neu5Gc ligands (Figure 2B) (71). Therefore, it can be expected that sIVIg cannot bind to murine CD22, and on basis of murine experiments it can therefore not be ruled out that sIVIg modulates immune responses in humans via binding to CD22.

In summary, while a role for DC-SIGN seems unlikely, other sialic acid-binding proteins have been identified that may alternatively mediate the anti-inflammatory properties of sIVIg, but currently there is only some functional evidence for involvement of CD22 in immunomodulation by sIVIg in humans. The fact that IL-33 and Th2 cytokines are elevated in plasma from IVIg-treated patients suggests that there is substantial overlap between the anti-inflammatory activity of IVIg in mice and men, but the molecular mechanism and the cellular source of stimulation of IL-33 production by IVIg needs further investigation.

## Stimulation of Regulatory T Cells by IVIg

Evidence has been accumulating that IVIg exerts its anti-inflammatory effects in experimental mice models also by stimulating expansion and suppressive function of CD4^+^Foxp3^+^ Tregs. Studies in disease models of ITP (72), EAE (17, 22, 73), antigen-driven allergic airway disease (62, 74, 75), Parkinson’s Disease (76), HSV-1-induced encephalitis (24), and allogeneic skin transplantation (77) have shown an indispensable role for Tregs in mediating the protective effects of IVIg treatment. Expansion of Tregs upon IVIg treatment was not an epiphenomenon associated with the dampening of an inflammatory reaction, as IVIg did not confer protection in studies in which Tregs were depleted prior to treatment (73, 77). A similar expansion of Tregs was observed in humans who were treated with IVIg for various diseases, such as Kawasaki disease (78, 79), Guillain Barré Syndrome (80, 81), rheumatoid arthritis (82) and eosinophilic granulomatosis with polyangiitis (83). In contrast, no expansion of Tregs was observed in a study on CVID patients that were treated with low-dose IVIg and had reduced Treg levels prior to treatment (84). Similar reduced Treg levels were found in another study on CVID patients after IVIg treatment, although levels of Tregs were not determined prior to treatment in these patients (85). We demonstrated that circulating Tregs in patients with various autoimmune diseases or immunodeficiencies are selectively activated upon high- but not low-dose IVIg therapy, as these cells showed increased FOXP3 and HLA-DR expression and enhanced suppressive capacity *ex vivo*, while T-helper cells were not affected (86). This finding was confirmed in a recent study showing enhanced *ex vivo* suppressive capacity of Tregs following IVIg treatment in Guillain-Barré Syndrome patients (81). Interestingly, our study did not show an increase of circulating Tregs upon IVIg treatment (86). Comparison of the various human studies reveals that expansion of Treg upon IVIg therapy was only observed in patients with inflammatory diseases in which the levels of Tregs were reduced prior to IVIg infusion, while Treg did not expand in patients without Treg deficit.

Besides an increase in Treg numbers, a concomitant decrease in Th17 cells has been observed upon IVIg administration. In murine models of EAE and collagen-induced arthritis, IVIg-induced expansion of Tregs while Th17 cell levels dropped (17, 87). In humans, there is similar evidence for reduction of Th17 cells after IVIg administration in Kawasaki disease (88) and Guillain Barré Syndrome patients (81). *In vitro* experiments suggest that IVIg inhibits cytokine-induced differentiation, amplification and function of Th17 cells from naive CD4^+^ T cells (89).

Collectively, expansion and enhanced suppressive activity of Tregs as well as reduction of pro-inflammatory Th17 cells seem to be common features of IVIg treatment in mice and humans. In humans high-dose IVIg therapy likely stimulates expansion of Treg only in conditions with deficit numbers of circulating Tregs before treatment. The latter observation differs from the findings in murine studies, which may be explained by the different compartments in which Treg are measured, i.e., Treg numbers in humans were determined in peripheral blood, and in mice in spleen, lymph nodes, and inflamed tissues.

Interestingly, Treg expansion and enhanced suppressive capacity may be related to induction of IL-33 production upon IVIg treatment. Three recent murine studies have shown that IL-33 directly stimulates CD4^+^Foxp3^+^ Treg expansion (90–92). In allogeneic heart transplant models IL-33 administration resulted in expansion of recipient Tregs in cardiac grafts and spleen and in prolonged allograft survival, while depletion of Tregs from recipients eliminated any therapeutic benefit from IL-33 therapy (90, 91). In a chronic colitis model, administration of IL-33 induced Treg proliferation *in vivo*, promoted Treg accumulation in the spleen and in inflamed tissues, and prevented loss of Foxp3 expression in the inflammatory environment (92). Stimulation of Tregs by IL-33 was dependent on expression of ST2, the IL-33 receptor, on Tregs (90, 92). *In vitro* experiments showed that IL-33 can serve as a cofactor in TGF-β-mediated Treg differentiation (92). However, there are no data yet to support a role of IL-33 in stimulation of Tregs by IVIg in humans.

**Figure 3:**
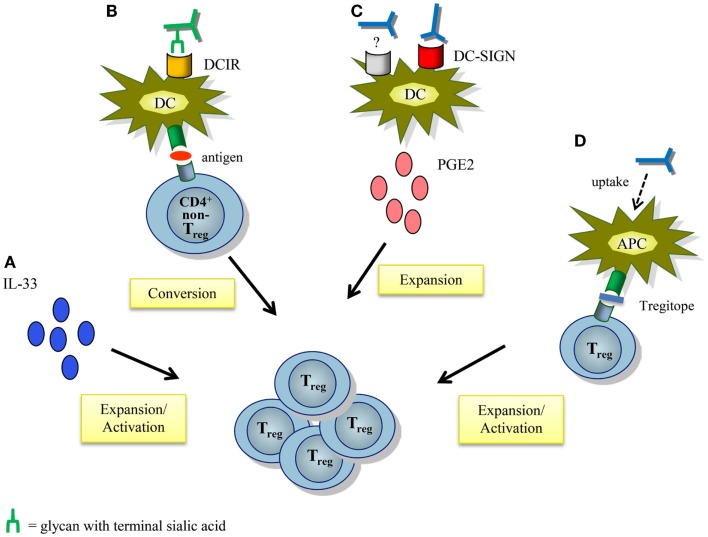
**Proposed mechanisms of action by which IVIg modulates regulatory T cells**. **(A)** IVIg-mediated IL-33 production induces Treg proliferation and activation. **(B)** IVIg induces antigen-specific Tregs by tolerogenic DCs from non-Treg CD4^+^ T cells by binding of sialylated IVIg to DCIR expressed on DCs. **(C)** IVIg induces prostaglandin E2 secretion by DCs, partly via DC-SIGN in a F(ab′)_2_-dependent manner. This leads to expansion of Tregs. The other “IVIg-receptor” involved in the secretion of PGE2 has yet to be identified. **(D)** Presentation of Treg-activating peptides derived from conserved epitopes of IgG (Tregitopes) by antigen-presenting cells activates and expands Tregs. APC, antigen-presenting cell; DCIR, dendritic cell immunoreceptor; PGE2, prostaglandin E2; Treg, regulatory T cell.

Several other mechanisms by which IVIg may modulate Treg function and expansion have been postulated, and these have been extensively reviewed elsewhere (Figure 3) (6, 93, 94). The contribution of IgG sialylation on Treg expansion has been recently addressed in several studies. As mentioned, in a murine model of antigen-driven allergic airway disease induction of antigen-specific Treg differentiation was dependent on binding of sIVIg to DCIR on DCs (62). In contrast, in HSE and EAE mouse models IgG sialylation was not required for functional activation of Tregs (22, 24). IVIg-induced expansion of human Tregs by moDCs *in vitro* was partially mediated by DC-SIGN in a F(ab′)_2_-dependent manner, but it is unknown whether IgG sialylation was involved (51). In addition, expansion and activation of Tregs upon recognition of specific IgG-derived peptides, called Tregitopes, that are presented by antigen-presenting cells, is sialylation-independent (95, 96). Collectively, sialylation is probably not required for the anti-inflammatory activity of IVIg via Tregs, but further study, especially in human settings, is warranted to unequivocally determine the contribution of IgG sialylation and the IL-33-Th2 cytokine cascade on IVIg-driven Treg expansion and function, both in antibody-mediated inflammatory diseases as well as in T-cell-mediated autoimmune pathologies.

## Toward Studying the Cellular and Molecular Pathways Required for IVIg-Mediated Immunosuppression in Humans

In Table 1, we have summarized the studies which we discussed above on the anti-inflammatory activity of IVIg in mice and humans. Despite the vast knowledge that has been gained on the anti-inflammatory activity of IVIg in mice, it is surprising how little we know of its immunosuppressive mechanisms-of-action in humans. The IL-33-Th2 cytokine cascade identified in mice is also activated in humans treated with IVIg, and results in reduced sensitivity of DCs to activation by ICs and IFN-γ, but there are significant differences in the cellular and molecular components of this pathway between mice and men. Most evidence shows no upregulation of inhibitory FcγRIIb, but instead downregulation of FcγRIIa expression on myeloid cells after IVIg-therapy in humans. Moreover, the hypothesis that Fc-sialylation is absolutely required for the anti-inflammatory effects of IVIg in mice has become controversial, and virtually no data supporting translation of this hypothesis to humans are available. In contrast, induction of Treg expansion and suppressive capacity by IVIg seems a common anti-inflammatory pathway exploited by IVIg in mice and men. However, the molecular mechanisms used by IVIg to initiate this anti-inflammatory cascades in humans are unresolved. Priority should therefore be given to investigate whether IgG glycoforms are critical components of anti-inflammatory activity of IVIg in humans. In addition, efforts to identify human IVIg-sensing molecules on human cells are warranted.

**Table 1:** **Summary of studies on the anti-inflammatory activities of IVIg in mice and humans**.

	Mice		Humans	
Anti-inflammatory activity of IVIG related to	Confirming evidence	Opposing evidence	Confirming evidence	Opposing evidence
**FcγRIIB/FcγRIIb**				
Functional role	ITP (8, 38)	ITP (15, 16)	None	None
	K/BxN arthritis (9, 10, 13, 14)	EAE (17)		
	EBA (14)			
	HSE (24)			

Enhanced expression	ITP (8)	None	HD: CIDP (32)	HD: autoimmune diseases (26)
	K/BxN arthritis (9)			LD: IgG deficiency (26)
	HSE (24)			LD: CVID (27)
				IVS: dendritic cells (26, 29)
				^a^HD: ITP (28)
				^a^HD: Kawasaki disease (29)
				^b^IVS: macrophages (31)

**IL-33**				
Functional role	K/BxN arthritis (13)	ITP (21)	None	None
Enhanced expression	K/BxN arthritis (13)	None	HD: autoimmune diseases (26)	None
			LD: IgG deficiency (26)	
			HD: rheumatic arthritis (36)	
			IVS: macrophages (26)	

**IL-4 and IL-13**				
Functional role	K/BxN arthritis (13)	ITP (21)	None	None
Enhanced expression	K/BxN arthritis (13)	None	HD: autoimmune diseases (26)	HD: rheumatoid arthritis (36)
			LD: IgG deficiency (26)	

**Basophils**				
Functional role	K/BxN arthritis (13)	K/BxN arthritis (23)	None	None
		CAI arthritis (23)		
		ITP (21)		
Expansion	K/BxN arthritis (13)	None	None	HD: rheumatoid arthritis (36)

**IgG Fc**	ITP (8)	EAE (17)	ITP (65, 66)	IVS: dendritic cells (51)
	K/BxN arthritis (9–13, 23)			IVS: monocytes (64)
	CAI arthritis (23)			

**Sialylation**	ITP (14, 21, 25)	ITP (19, 20)	IVS: B cells (63)	None
	K/BxN arthritis (10–14, 25)	K/BxN arthritis (23)		
	CAI arthritis (25)	CAI arthritis (23)		
	EBA (14, 25)	EAE (22)		
	Allergic airway disease (62)	HSE (24)		

**IVIg-binding proteins**				
SIGN-R1/DC-SIGN	ITP (21)	ITP (14)	IVS: dendritic cells (51)	IVS: macrophages (26)
	K/BxN arthritis (10, 13, 14)			IVS: dendritic cells (36)
	EBA (14)			IVS: splenocytes (26, 36)
CD22	None	^c^ITP (69)	IVS: B cells (63)	None
		^c^K/BxN arthritis (69)		
DCIR	Allergic airway disease (62)	None	None	None

**Regulatory T cells**				
Expansion	ITP (72)	None	HD: Kawasaki disease (78, 79)	HD: autoimmune diseases (86)
	EAE (17, 22, 73)		HD: Guillain Barré (80, 81)	LD: IgG deficiency (86)
	Allergic airway disease (62, 74, 75)		HD: rheumatoid arthritis (82)	LD: CVID (84)
	Parkinson’s disease (76)		HD: eosinophilic granulomatosis (83)	
	HSE (24)		IVS (51, 89)	
	CAI arthritis (87)			
Enhanced suppressive capacity	EAE (73)	None	HD: autoimmune diseases (86)	None
	Allergic airway disease (75)		HD: Guillain Barré (81)	
	Skin-allograft (77)			

*HD, high dose IVIg; LD, low-dose IVIg; IVS, *in vitro* studies on human immune cells; ITP, immune thrombocytopenic purpura model; EBA, autoimmune skin-blistering epidermolysis bullosa acquisita pemphigus model; K/BxN arthritis, K/BxN serum-induced arthritis model; CAI arthritis, collagen-antibody-induced arthritis model; EAE, experimental autoimmune encephalomyelitis model; HSE, HSV-1-induced encephalitis model; CIDP, chronic inflammatory demyelinating polyneuropathy; CVID, common variable immune deficiency*.

*^a^The antibody used in these studies is not suitable to detect the surface expression of FcγRIIb*.

*^b^IVIg concentration used in this study was at least 100-fold lower than the anti-inflammatory IVIg concentration of 10 mg/ml*.

*^c^CD22^−/−^ mice are not suitable to determine whether the anti-inflammatory activity of IVIg is mediated via human CD22 (see Alternative IVIg-Sensing Molecules)*.

Although we value the mechanistic insights that have been gained on the working mechanisms of IVIg in mice, we recommend, in light of the biological differences between mice and men, studying the immunomodulatory pathways of IVIg in humans using *ex vivo* measurements, as well as *in vitro* studies on human cells. We advocate for the initiation of large, multicenter trials on patients with various indications to most effectively answer how IVIg modulates the immune system in humans *in vivo* at a cellular and molecular level, ideally with system biology approaches. Only then can we truly dissect which anti-inflammatory mechanisms are activated by IVIg and which components within IVIg are essential to gain potent anti-inflammatory responses that are disease- or risk group-specific. Alternatively, to address causality, studies should be initiated using immunodeficient mice reconstituted with a human immune system, although it is unclear whether current humanized mice disease models are adequate enough to mimic what occurs upon IVIg treatment in humans (97). In the light of the growing demand for IVIg, concomitant with the predicted shortage of human plasma in the future and the high costs of IVIg therapy, it is of utmost importance to unravel the molecular interactions between IVIg and the human immune system, as such knowledge may enable the design of biologicals or small molecule drugs that mimic the anti-inflammatory effects of IVIg.

## Author Contributions

All the authors were involved in designing, drafting and revising the manuscript. In addition, the authors have all given final approval for the manuscript to be published, and agree to its accuracy and integrity.

## Conflict of Interest Statement

The authors declare that the research was conducted in the absence of any commercial or financial relationships that could be construed as a potential conflict of interest.
